# Lactic acid bacterium, *Lactobacillus paracasei* KW3110, suppresses inflammatory stress-induced caspase-1 activation by promoting interleukin-10 production in mouse and human immune cells

**DOI:** 10.1371/journal.pone.0237754

**Published:** 2020-08-17

**Authors:** Takahiro Yamazaki, Konomi Ohshio, Miho Sugamata, Yuji Morita

**Affiliations:** KIRIN Central Research Institute, Kirin Holdings Company, Limited, Kanagawa, Yokohama, Japan; University of the Pacific, UNITED STATES

## Abstract

A strain of lactic acid bacteria, *Lactobacillus paracasei* KW3110 (KW3110), activates M2 macrophages with anti-inflammatory reactions and mitigates aging-related chronic inflammation and blue-light exposure-induced retinal inflammation in mice. However, the mechanism underlying the anti-inflammatory effects of KW3110 remains unclear. In this study, we investigated the anti-inflammatory effects of KW3110 using both mouse and human immune cells and evaluated the suppressive effect of KW3110 on the inflammatory reactions of the cells stimulated with lipopolysaccharide and adenosine 5′-triphosphate (LPS/ATP). KW3110 treatment induced anti-inflammatory cytokine interleukin (IL)-10 production in the supernatants of murine macrophage-like cells, J774A.1, and suppressed IL-1β production in the supernatants of LPS/ATP-stimulated cells. The influence of KW3110 on the production of these cytokines was inhibited by pre-treatment with phagocytosis blocker or transfection with siRNAs for IL-10 signaling components. KW3110 treatment also suppressed activation of caspase-1, an active component of inflammasome complexes, in LPS/ATP-stimulated J774A.1 cells, and its effect was inhibited by transfection with siRNAs for IL-10 signaling components. In addition to the effects of KW3110 on J774A.1 cells, KW3110 treatment induced IL-10 production in the supernatants of human monocytes, and KW3110 or IL-10 treatment suppressed caspase-1 activation and IL-1β production in the supernatants of LPS/ATP-stimulated cells. These results suggest that KW3110 suppresses LPS/ATP stimulation-induced caspase-1 activation and IL-1β production by promoting IL-10 production in mouse and human immune cells. Our findings reveal a novel anti-inflammatory mechanism of LAB and the effect of KW3110 on caspase-1 activation is expected to contribute to constructing future preventive strategies for inflammation-related disorders using food ingredients.

## Introduction

The immune response is a fundamental system developed in animals to combat endogenous or exogenous antigens or other stresses. Inflammation is an important process in the immune response as it eliminates infectious factors and damaged cells and tissues, initiating tissue repair. Various stresses, such as some infections or aging, can cause excessive or chronic inflammation, leading to serious disorders or diseases and this is affecting more and more people [1–4]. Interventions to prevent the conditions related to those stresses are necessary.

Food ingredients with immune modulating functions have been studied as one of the strategies to prevent inflammation-related disorders or diseases [5–7]. Lactic acid bacteria (LAB) are well known sources of probiotics and paraprobiotics which improve gut barrier function and the immune system. Some LAB strains are reported to ameliorate several inflammation-related disorders, such as allergies and metabolic disorders [8, 9]; however, the mechanism of action is not completely understood.

We previously reported that a specific strain of LAB, *Lactobacillus paracasei* KW3110 (KW3110), suppresses excessive inflammation including dermatitis in mice and humans [8, 10–12]. KW3110 was shown to activate M2 macrophages with anti-inflammatory reactions, and induce the production of anti-inflammatory cytokine interleukin (IL)-10 [13]. In addition, KW3110 mitigated aging-related chronic inflammation and blue-light exposure-induced retinal inflammation in mice, at least partially through activating immune cells and reducing pro-inflammatory cytokine production, such as IL-1β [13, 14]; however, the mechanism underlying the anti-inflammatory effects of KW3110 remains unclear.

To investigate the detailed site of action of the anti-inflammatory effects of KW3110, we performed in vitro experiments with murine macrophage-like cell line, J774A.1 which is a well-established cell line producing pro- or anti-inflammatory cytokines in response to various stimulations [15, 16]. Since human monocytes have been used to investigate inflammatory reactions and their mechanism in human macrophages, we also used a human cell line to determine whether the mechanisms of anti-inflammation are preserved between murine and human cells [17, 18]. Our study demonstrated that KW3110 suppressed inflammatory stress-induced activation of an active component of inflammasome complexes, caspase-1, and production of IL-1β by promoting IL-10 production in murine macrophages and human monocytes. These findings reveal a novel anti-inflammatory mechanism of LAB and are expected to contribute to constructing future preventive strategies for inflammation-related disorders using food ingredients.

## Materials and methods

### Materials

KW3110 was maintained at Koiwai Dairy Products Co., Ltd (Tokyo, Japan). KW3110 was grown at 37 ˚C for 48 h, heat-killed at 100 ˚C, lyophilized, and suspended in PBS [12]. The list of purchased items includes: ultrapure lipopolysaccharide from *E*. *coli* 0111:B4 strain (LPS) (Invivogen, San Diego, CA, USA), adenosine 5′-triphosphate (ATP) and cytochalasin D (Sigma, St. Louis, MO, USA), recombinant mouse and human IL-10 proteins (R&D Systems, MN, USA), anti-mouse caspase-1 (p20) (Adipogen Life Sciences (San Diego, CA, USA); catalog number: AG-20B-0042-C100; mouse-monoclonal antibody), anti-mouse IL-1β (catalog number: 12507S; rabbit-monoclonal antibody) and anti-mouse β-actin (catalog number: 3700; mouse-monoclonal antibody) (Cell Signaling Technology, Danvers, MA, USA) and secondary antibodies, anti-mouse IgG horseradish peroxidase-linked whole antibody from sheep (catalog number: NA931) and anti-rabbit IgG horseradish peroxidase-linked whole antibody from donkey (catalog number: NA934) (GE Healthcare, Chicago, IL, USA).

### Cell culture

The J774A.1 cell line, derived from ascites obtained from an adult female mouse with reticulum cell sarcoma [19] (ATCC (Manassas, VA, USA); catalog number: TIB-67), was maintained in Dulbecco’s modified Eagle medium with 10% fetal bovine serum, 100 U/mL of penicillin, and 100 μg/mL of streptomycin (Gibco, Grand Island, NY, USA) at 37 ˚C in 5% CO_2_ /air humidity.

### Preparation of human monocytes

Human peripheral blood mononuclear cells from healthy donors were purchased from iQ Biosciences (Berkeley, CA, USA; catalog number: IQB-PBMC103). Written consent was obtained from all donor volunteers participating in a donor program that was approved by Western Institutional Review Board, and de-identification was performed. Monocytes were isolated from the mononuclear cells using a classical monocyte isolation kit (Miltenyi Biotec, Sunnyvale, CA, USA), according to the manufacturer’s instructions.

### Determination of cytokine production

**Fig 1:**
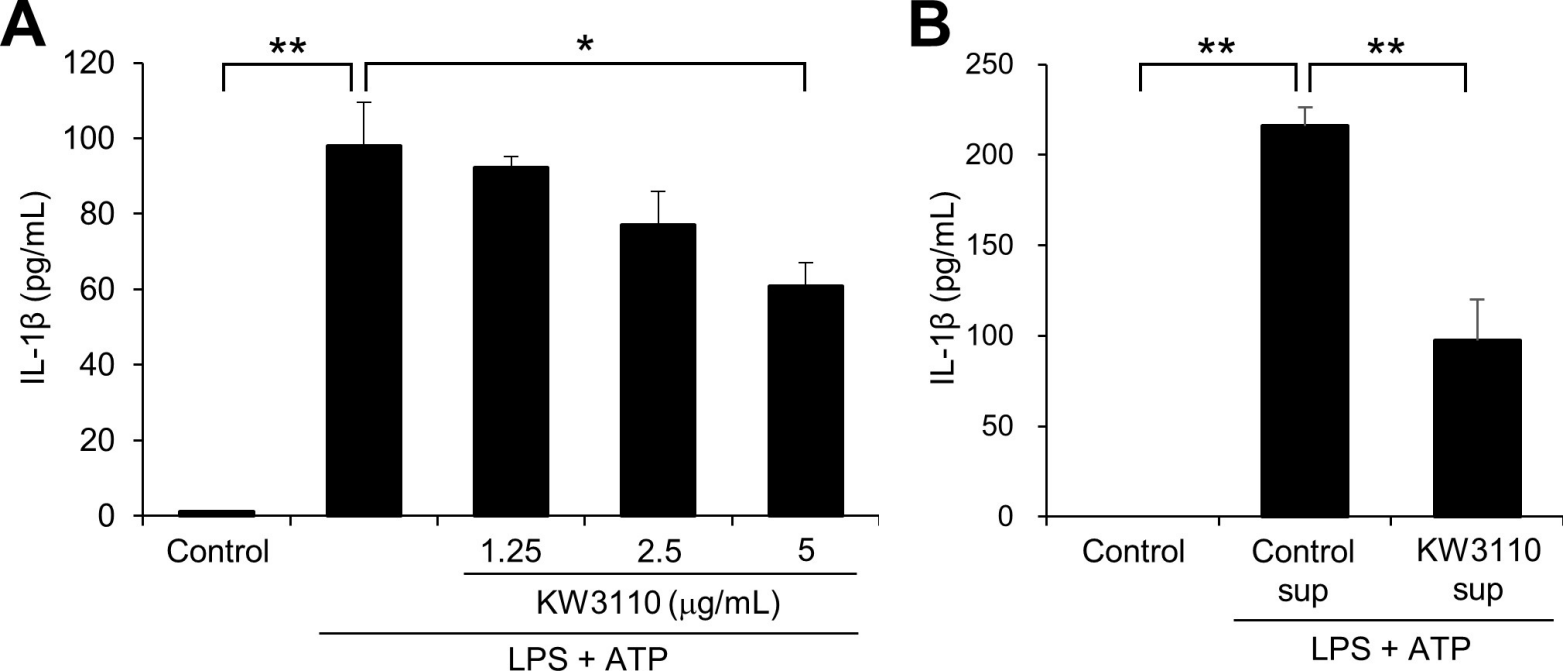
KW3110 treatment suppresses IL-1β production in LPS/ATP-stimulated J774A.1 cells. (A) J774A.1 cells were treated with KW3110 (1.25, 2.5, or 5 μg/mL) for 24 h and treated with LPS (10 μg/mL) for 4 h and ATP (2 mM) for 1 h. IL-1β levels in the supernatant were measured by ELISA. (B) The supernatant from KW3110-treated J774A.1 cells was transferred to non-treated cells which were then treated with LPS (10 μg/mL) for 4 h and ATP (2 mM) for 1 h. IL-1β levels in the supernatant were measured by ELISA. Control sup; supernatant from non-treated J774A.1 cells, KW3110 sup; supernatant from KW3110-treated J774A.1 cells. Values indicate means ± SEM (triplicate data). Statistical differences were analyzed by ANOVA followed by Tukey’s test (*, *P* < 0.05; **, *P* < 0.01).

Cells were seeded and incubated overnight in 24-well plates (J774A.1 cells, 1 × 10^5^ cells/well; human monocytes, 2.5 × 10^5^ cells/well), and treated with KW3110 (1.25–5 μg/mL for J774A.1 cells and 100 μg/mL for human monocytes) for 24 h at 37 ˚C. To measure IL-1β, the cells were treated with 1 μg/mL of IL-10 or primed with 10 μg/mL of LPS for 4 h, and stimulated with 2 mM ATP for 1 h. For the supernatant transferal experiments, in Fig 1B, the supernatants collected from KW3110-treated J774A.1 cells were transferred to other cells which were then primed with 10 μg/mL of LPS for 4 h and stimulated with 2 mM ATP for 1 h. For the phagocytosis blocking experiments, 1 μg/mL of cytochalasin D was added 30 min prior to KW3110 treatment of J774A.1 cells. The supernatants were then collected and centrifuged at 5,000 rpm for 2 min. Cytokine levels were measured with commercial enzyme-linked immunosorbent assay (ELISA) kits (mouse and human IL-10, BD Biosciences, San Jose, CA, USA; mouse IL-1β, eBioscience, San Diego, CA, USA; human IL-1β, R&D Systems, Minneapolis, MN, USA), according to the manufacturer’s instructions.

### Western blotting

J774A.1 cells were seeded and incubated overnight in 24-well plates (1 × 10^5^ cells/well), and treated with 5 μg/mL of KW3110 in Opti-MEM with 1% fetal bovine serum (Gibco, Grand Island, NY, USA) for 24 h at 37 ˚C. The cells were then primed with 10 μg/mL of LPS for 4 h, and stimulated with 2 mM ATP for 1 h. After stimulation, cells were lysed with radioimmunoprecipitation assay buffer and whole cell lysates were prepared and then boiled in sodium dodecyl sulfate (SDS) sample buffer for 5 min at 95 ˚C. Supernatants were precipitated with methanol/chloroform and the pellets were lysed in the SDS sample buffer and then boiled for 5 min at 95 ˚C. SDS-polyacrylamide gel electrophoresis-separated samples were transferred to polyvinylidene difluoride membranes and immunoblotted with primary antibodies (1:1000 dilution) overnight at 4 ˚C, followed by secondary antibodies (1:10000 dilution) for 1 h at room temperature. The membranes were scanned with an ImageQuant LAS 4000 (GE Healthcare, Chicago, IL, USA).

### Caspase-1 activity assay

Cells were seeded and incubated overnight in 24-well plates (J774A.1 cells, 1 × 10^5^ cells/well; human monocytes, 2.5 × 10^5^ cells/well) before treatment with KW3110 (5 μg/mL for J774A.1 cells and 100 μg/mL for human monocytes) for 24 h at 37 ˚C. The cells were then treated with 1 μg/mL of IL-10 or primed with 10 μg/mL of LPS for 4 h, and stimulated with 2 mM ATP for 1 h. These supernatants were collected and centrifuged at 5,000 rpm for 2 min. Caspase-1 activity levels were measured with a Caspase-Glo 1 Inflammasome assay kit (Promega, Madison, WI, USA), according to the manufacturer’s instructions.

### Quantitative real-time RT-PCR

J774A.1 cells were seeded and incubated overnight in 24-well plates (3 × 10^5^ cells/well) before treatment with 5 μg/mL of KW3110 for 6 h at 37 ˚C. Total RNA was extracted with an RNeasy mini kit (QIAGEN, Venlo, Netherlands) and reverse-transcribed with a SuperScript IV First-Strand Synthesis System (Invitrogen, Waltham, MA, USA). Quantitative RT‑PCR was performed with TB Green Premix Ex Taq (Takara Bio, Shiga, Japan) and a LightCycler 480 (Roche Diagnostics, Rotkreuz, Switzerland). The PCR conditions were as follows: 2‑step cycling, 95 ˚C for 10-sec hold, 45 cycles of 95 ˚C for 5 sec and 60 ˚C for 20 sec. All values were normalized to *GAPDH* expression. Specific forward and reverse primer pairs were: *GAPDH* forward, 5'‑TGTGTCCGTCGTGGATCTGA‑3' and reverse, 5'‑TTGCTGTTGAAGTCGCAGGAG‑3'; *IL-10* forward, 5‑GCCAGAGCCACATGGTCCTA‑3' and reverse, GATAAGGCTTGGCA ACCCAAGTAA‑3'; *IL-10 receptor α* forward, 5‑CCAGTCTGAGAGCACCTACTATGAA‑3' and reverse, 5’-CCAGGGTGAACGTTGTGAGA‑3'.

### siRNA and transfection

Small interfering RNAs (siRNAs) were all siGENOME SMARTpool siRNA (Dharmacon, Lafayette, CO, USA). Non-targeting siRNA (*siNT*, negative control), mouse *IL-10* siRNA (*siIL-10*), and mouse *IL-10 receptor α* siRNA (*siIL-10R*) were used in this study. J774A.1 cells were seeded and incubated in 24-well plates (3 × 10^5^ cells/well) and transfected with 100 nM siRNAs using DharmaFECT 1 transfection reagent (Dharmacon, Lafayette, CO, USA) for 24 h before KW3110 treatment.

### Statistical analysis

Values indicate means ± SEM. Statistical differences were analyzed by unpaired *t*-test or analysis of variance (ANOVA) followed by Dunnett’s or Tukey’s test. *P* values < 0.05 were considered statistically significant. Experiments of evaluating cytokine production and caspase-1 activity were performed at least twice independently.

## Results

### KW3110 treatment suppresses IL-1β production in LPS/ATP-stimulated J774A.1 cells

We investigated how KW3110 suppresses the inflammatory response in macrophages using a murine macrophage-like cell line, J774A.1. IL-1β levels were significantly increased in the supernatant from LPS/ATP-stimulated J774A.1 cells when compared with the control (*P* < 0.001; Fig 1A); KW3110 pre-treatment of the cells dose-dependently decreased IL-1β levels (5 μg/mL, *P* = 0.027; Fig 1A). In addition, IL-1β levels were significantly decreased in the supernatant from LPS/ATP-stimulated cells after treatment with the supernatant from KW3110-treated cells (*P* = 0.003; Fig 1B).

### KW3110 treatment suppresses IL-1β production in LPS/ATP-stimulated J774A.1 cells through IL-10 signaling

**Fig 2:**
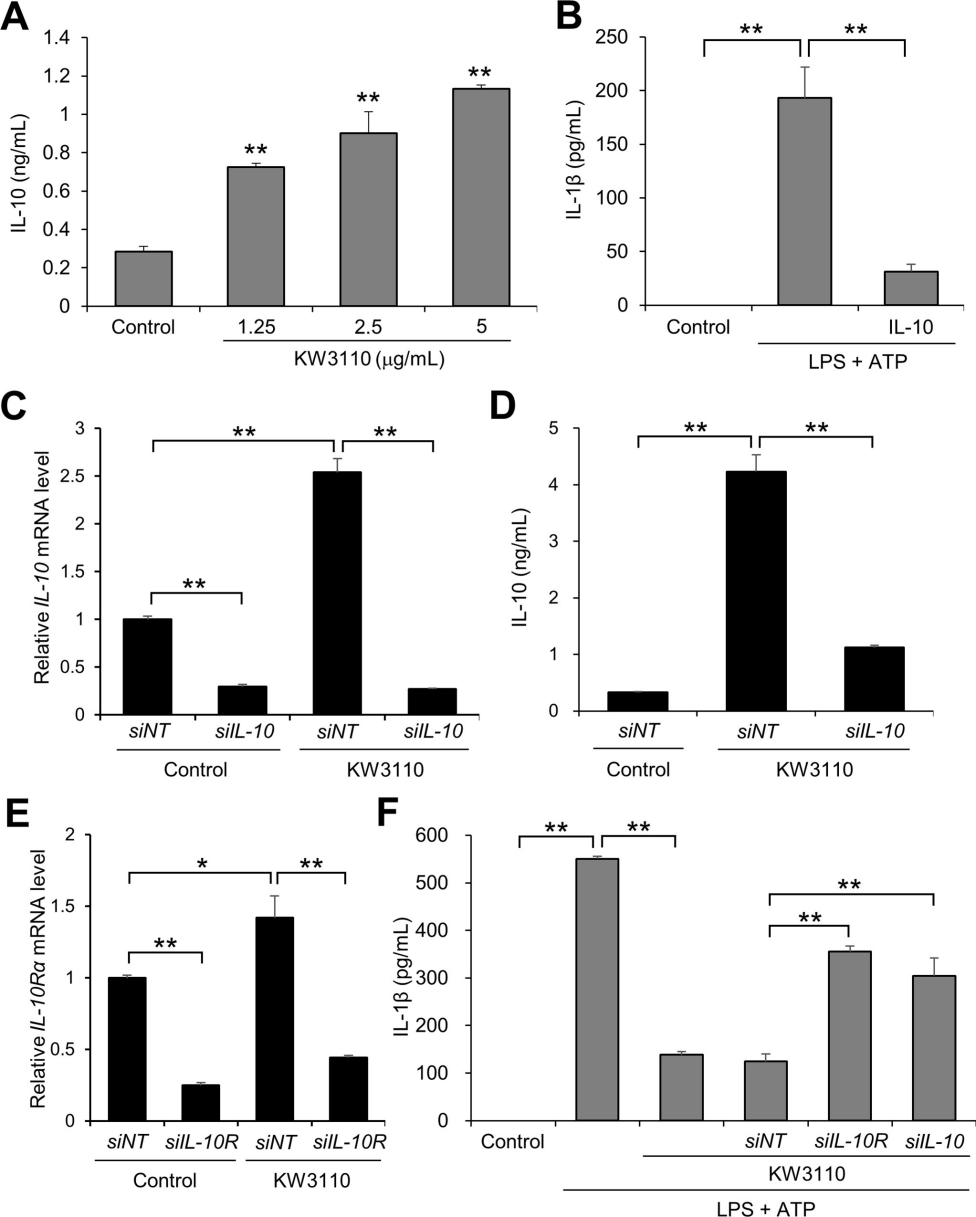
Knockdown of IL-10 signaling components reduces KW3110-induced IL-1β suppression in LPS/ATP-stimulated J774A.1 cells. (A) J774A.1 cells were treated with KW3110 (1.25, 2.5, or 5 μg/mL) for 24 h. IL-10 levels in the supernatant were measured by ELISA. (B) The cells were treated with IL-10 (1 μg/mL) and LPS (10 μg/mL) for 4 h and ATP (2 mM) for 1 h. IL-1β levels in the supernatant were measured by ELISA. (C) Total RNA from cells transfected with *IL-10* siRNA and treated with KW3110 (10 μg/mL) for 6 h was extracted and reverse-transcribed. Quantitative RT-PCR was performed to amplify mouse *GAPDH* and *IL-10*. The *IL-10* values were normalized to the expression of *GAPDH*. (D, E) Cells were transfected with *IL-10* or *IL-10 receptor α* siRNAs and treated with KW3110 (10 μg/mL) for 24 h. IL-10 levels in the supernatant were measured by ELISA for (D). The cells were then treated with LPS (10 μg/mL) for 4 h and ATP (2 mM) for 1 h. IL-1β levels in the supernatant were measured by ELISA for (E). *siNT*; siRNA for non-targeting, *siIL-10*; siRNA for *IL-10*, *siIL-10R*; siRNA for *IL-10 receptor* α. Values indicate means ± SEM (triplicate data). Statistical differences were analyzed by ANOVA followed by Dunnett’s test for (A) and Tukey’s test for (B-E) (**, *P* < 0.01).

The supernatant transferal experiment (Fig 1B) suggested that molecules released from KW3110-treated J774A.1 cells suppressed IL-1β production in LPS/ATP-stimulated cells. KW3110 induces IL-10 production in M2 macrophages; therefore, we hypothesized that KW3110 suppresses the inflammatory response in macrophages through IL-10 induction [13, 20]. KW3110 treatment dose-dependently increased IL-10 levels in J774A.1 cell supernatant (1.25 μg/mL, *P* = 0.002; 2.5 μg/mL, *P* < 0.001; 5 μg/mL, *P* < 0.001; Fig 2A). Treatment with recombinant IL-10 protein significantly decreased IL-1β levels in supernatant from LPS/ATP-stimulated cells (*P* = 0.001; Fig 2B). To investigate the contribution of IL-10 signaling to KW3110-induced suppression of IL-1β production in LPS/ATP-stimulated cells, we performed genetic knockdown experiments with siRNAs targeting IL-10 signaling components. We confirmed that both mRNA and protein levels of IL-10 were significantly decreased in KW3110-treated cells by transfection with siRNA for *IL-10* (*P* < 0.001; Fig 2C) (*P* < 0.001; Fig 2D), and that mRNA level of *IL-10 receptor α* was significantly decreased in KW3110-treated cells by transfection with siRNA for *IL-10 receptor α* (*P* < 0.001; Fig 2E). The suppressive effect of KW3110 on IL-1β production in LPS/ATP-stimulated cells was significantly inhibited by transfection with siRNAs for *IL-10* or *IL-10 receptor α* (*IL-10*, *P* < 0.001; *IL-10 receptor* α, *P* < 0.001; Fig 2E).

### Phagocytosis is essential for KW3110-induced IL-10 production and IL-1β suppression in J774A.1 cells

**Fig 3:**
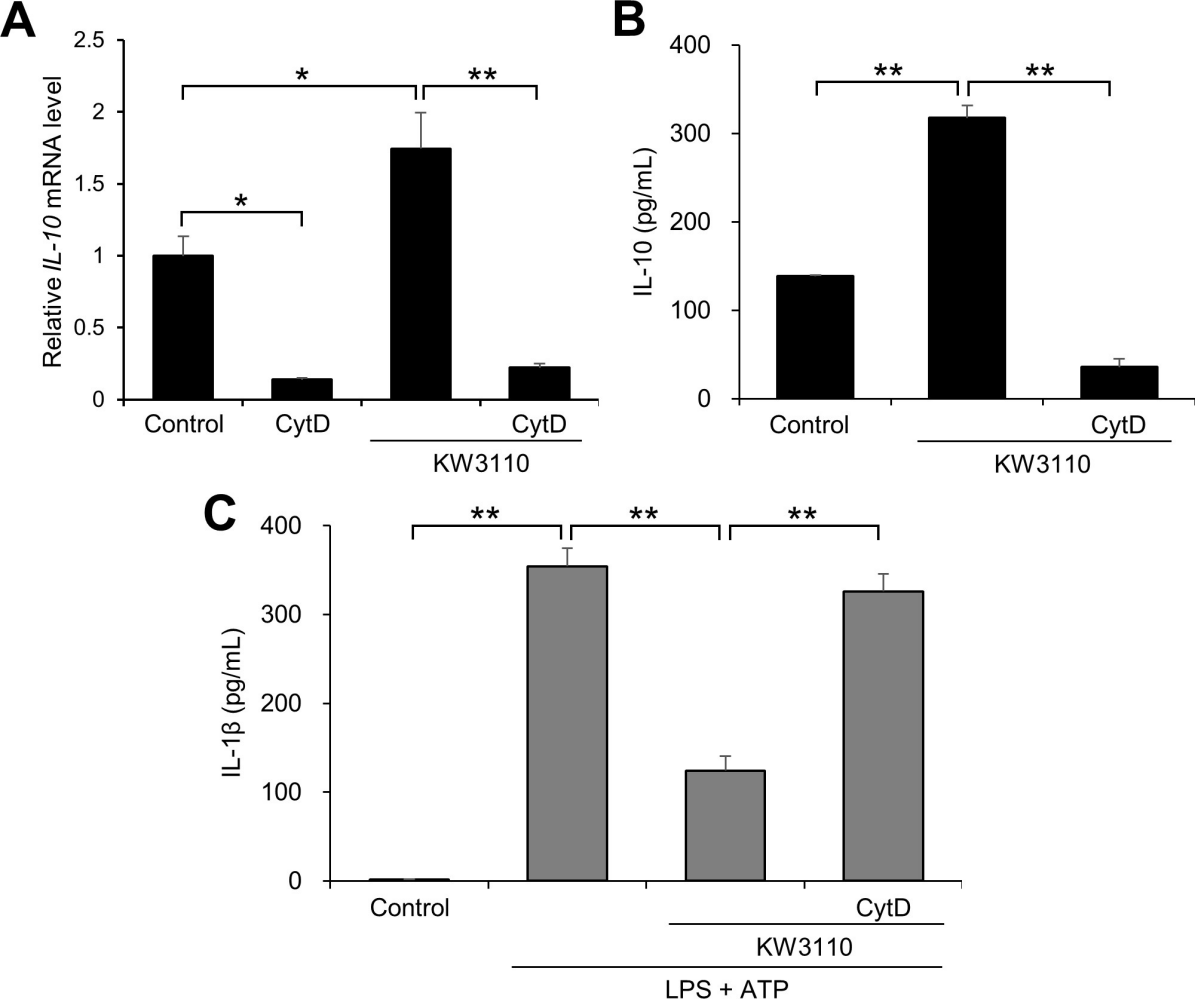
Effects of phagocytosis blocker on KW3110-induced IL-10 production and IL-1β suppression in J774A.1 cells. (A) Total RNA from J774A.1 cells treated with cytochalasin D (1 μg/mL) for 30 min and KW3110 (5 μg/mL) for 6 h was extracted and reverse-transcribed. Quantitative RT-PCR was performed to amplify mouse *GAPDH* and *IL-10*. *IL-10* values were normalized to the expression of *GAPDH*. (B, C) The cells were treated with cytochalasin D (1 μg/mL) for 30 min and KW3110 (5 μg/mL) for 24 h. IL-10 levels in the supernatant were measured by ELISA for (B). The cells were then treated with LPS (10 μg/mL) for 4 h and ATP (2 mM) for 1 h. IL-1β levels in the supernatant were measured by ELISA for (C). CytD; cytochalasin D. Values indicate means ± SEM (triplicate data). Statistical differences were analyzed by ANOVA followed by Tukey’s test (**, *P* < 0.01).

Macrophages detect and phagocytose microbes including LAB, producing pro- or anti-inflammatory cytokines [21, 22]. To investigate whether KW3110 activates macrophages and promotes IL-10 production through phagocytosis, we used a phagocytosis blocker, cytochalasin D, before treatment of J774A.1 cells with KW3110. Cytochalasin D pre-treatment significantly decreased both mRNA and protein levels of IL-10 in KW3110-treated cells (*P* < 0.001; Fig 3A) (*P* < 0.001; Fig 3B). Furthermore, the suppressive effect of KW3110 on IL-1β production in the supernatant from LPS/ATP-stimulated cells was significantly inhibited by cytochalasin D pre-treatment (*P* < 0.001; Fig 3C).

### KW3110 suppresses the active form of caspase-1 expression in LPS/ATP-stimulated J774A.1 cells through IL-10 signaling

**Fig 4:**
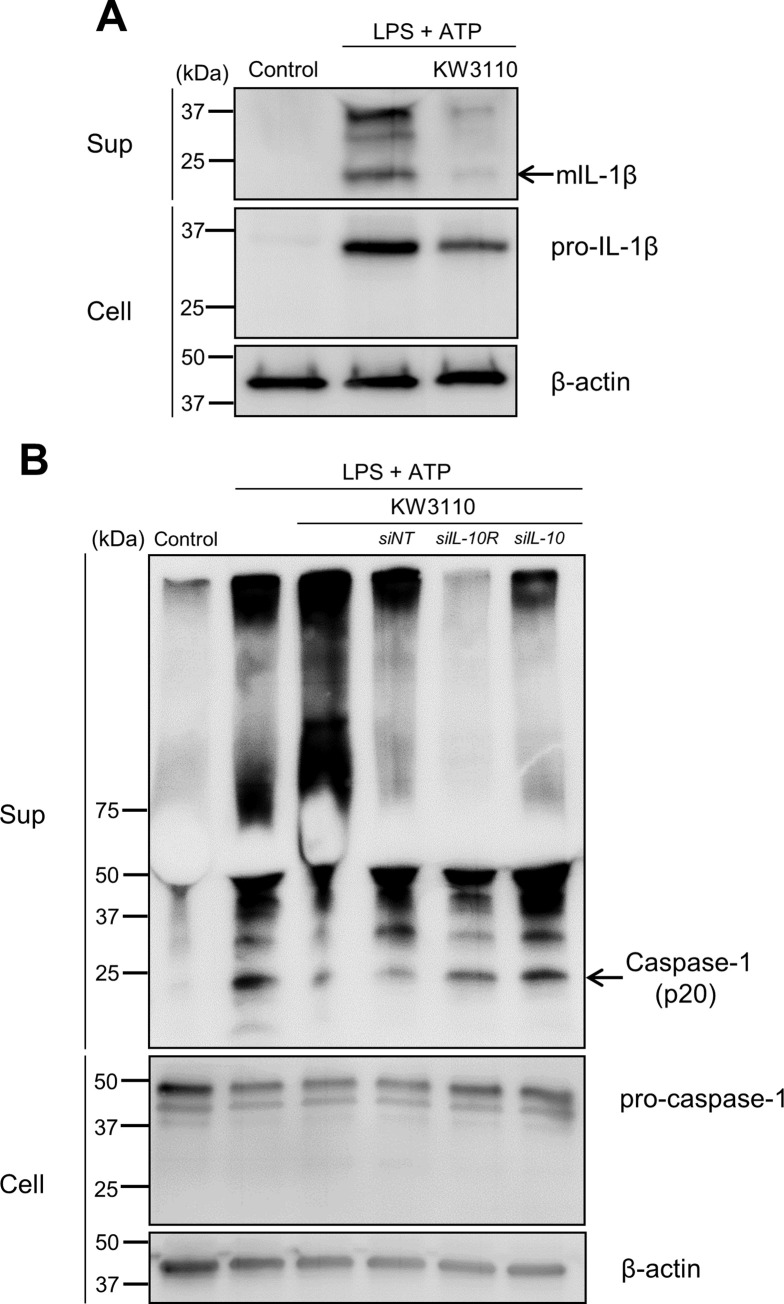
KW3110 treatment suppresses active form of caspase-1 expression in LPS/ATP-stimulated J774A.1 cells through IL-10 signaling. (A) J774A.1 cells were treated with KW3110 (5 μg/mL) for 24 h, and treated with LPS (10 μg/mL) for 4 h and ATP (2 mM) for 1 h. The supernatant and cell lysate were analyzed by western blotting to detect matured IL-1β, pro-IL-1β, and β-actin. (B) The cells were transfected with *IL-10* or *IL-10 receptor α* siRNAs and treated with KW3110 (10 μg/mL) for 24 h, and then treated with LPS (10 μg/mL) for 4 h and ATP (2 mM) for 1 h. The supernatant and cell lysate were analyzed by western blotting to detect caspase-1 (p20), pro-caspase-1, and β-actin. The black arrows are pointing to bands representing matured IL-1β or caspase-1 (p20). All blot images are spliced along with black lines. Sup; supernatant, Cell; cell lysate, *siNT*; siRNA for non-targeting, *siIL-10*; siRNA for *IL-10*, *siIL-10R*; siRNA for *IL-10 receptor α*, mIL-1β; mature IL-1β.

We investigated the detailed mechanism of how KW3110 suppresses IL-1β production in LPS/ATP-stimulated J774A.1 cells. The suppression of mature IL-1β expression in the supernatant from KW3110- and LPS/ATP-stimulated cells was confirmed by western blotting, whereas pro-IL-1β expression levels in the cell lysates was not affected by KW3110 treatment (Fig 4A). Since IL-1β maturation is promoted by the activation of inflammatory cysteine protease caspase-1 in inflammasome complexes, we investigated whether KW3110 suppresses caspase-1 activation in macrophages. Levels of the active form of caspase-1 were increased in the supernatant from LPS/ATP-stimulated cells when compared with the control, whereas KW3110 pre-treatment of the cells suppressed caspase-1 levels (Fig 4B). Moreover, the suppressive effect of KW3110 on the active form of caspase-1 was inhibited by transfection with *IL-10* or *IL-10 receptor α* siRNAs (Fig 4B).

### KW3110 or IL-10 treatment suppresses caspase-1 activation in LPS/ATP-stimulated J774A.1 cells

**Fig 5:**
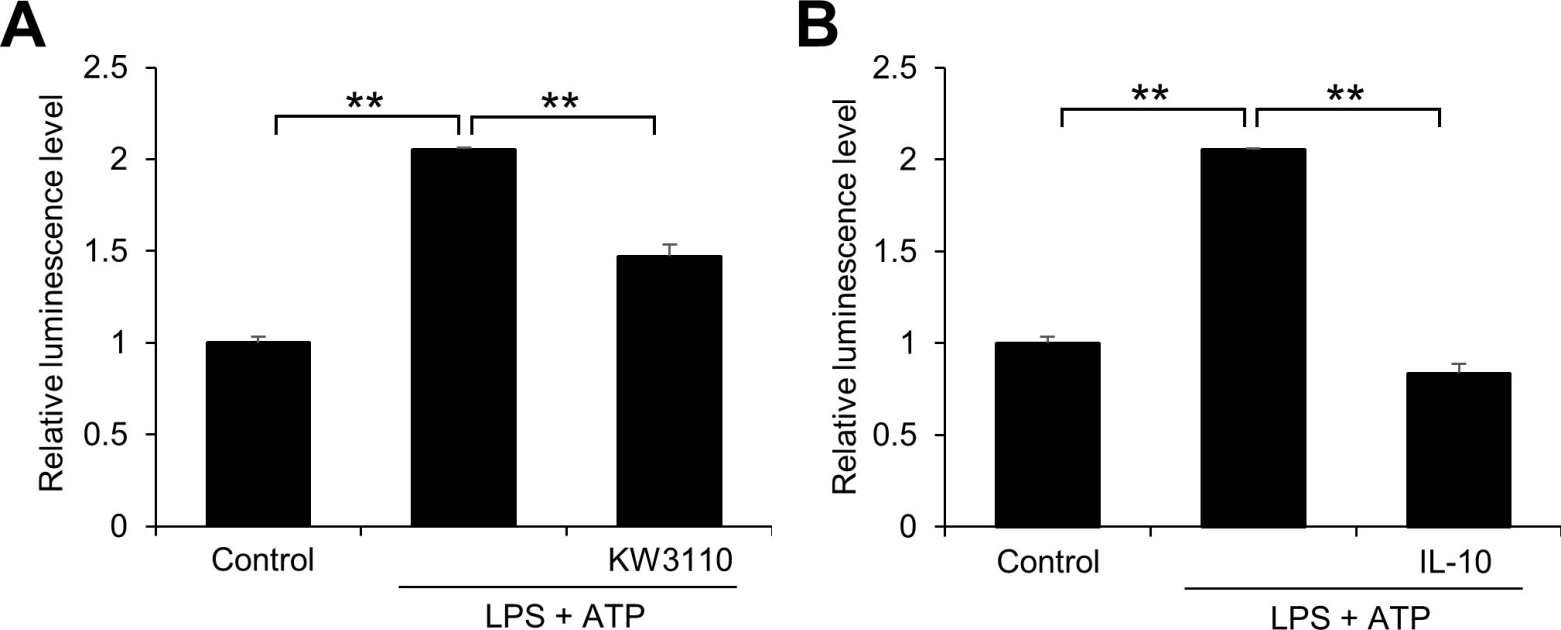
KW3110 or IL-10 treatment suppresses caspase-1 activation in LPS/ATP-stimulated J774A.1 cells. (A) J774A.1 cells were treated with KW3110 (5 μg/mL) for 24 h, and treated with LPS (10 μg/mL) for 4 h and ATP (2 mM) for 1 h. (B) Cells were treated with IL-10 (1 μg/mL) and LPS (10 μg/mL) for 4 h and ATP (2 mM) for 1 h. Caspase-Glo 1 inflammasome assays measured caspase-1 activity in the supernatants from treated cells. Values indicate means ± SEM (triplicate data). Statistical differences were analyzed by ANOVA followed by Tukey’s test (**, *P* < 0.01).

To confirm that KW3110 treatment suppresses caspase-1 activity in LPS/ATP-stimulated J774A.1 cells, we performed Caspase-Glo 1 inflammasome assays to quantitatively measure caspase-1 activities. LPS/ATP stimulation of J774A.1 cells significantly increased caspase-1 activity levels in the cell supernatant (*P* < 0.001; Fig 5A), and the increase was significantly suppressed by KW3110 pre-treatment (*P* < 0.001; Fig 5A). In addition, IL-10 treatment of LPS/ATP-stimulated cells significantly decreased caspase-1 activity levels in the supernatant from cells (*P* < 0.001; Fig 5B).

### Effects of KW3110 treatment on caspase-1 activity and IL-1β production in human monocytes

**Fig 6:**
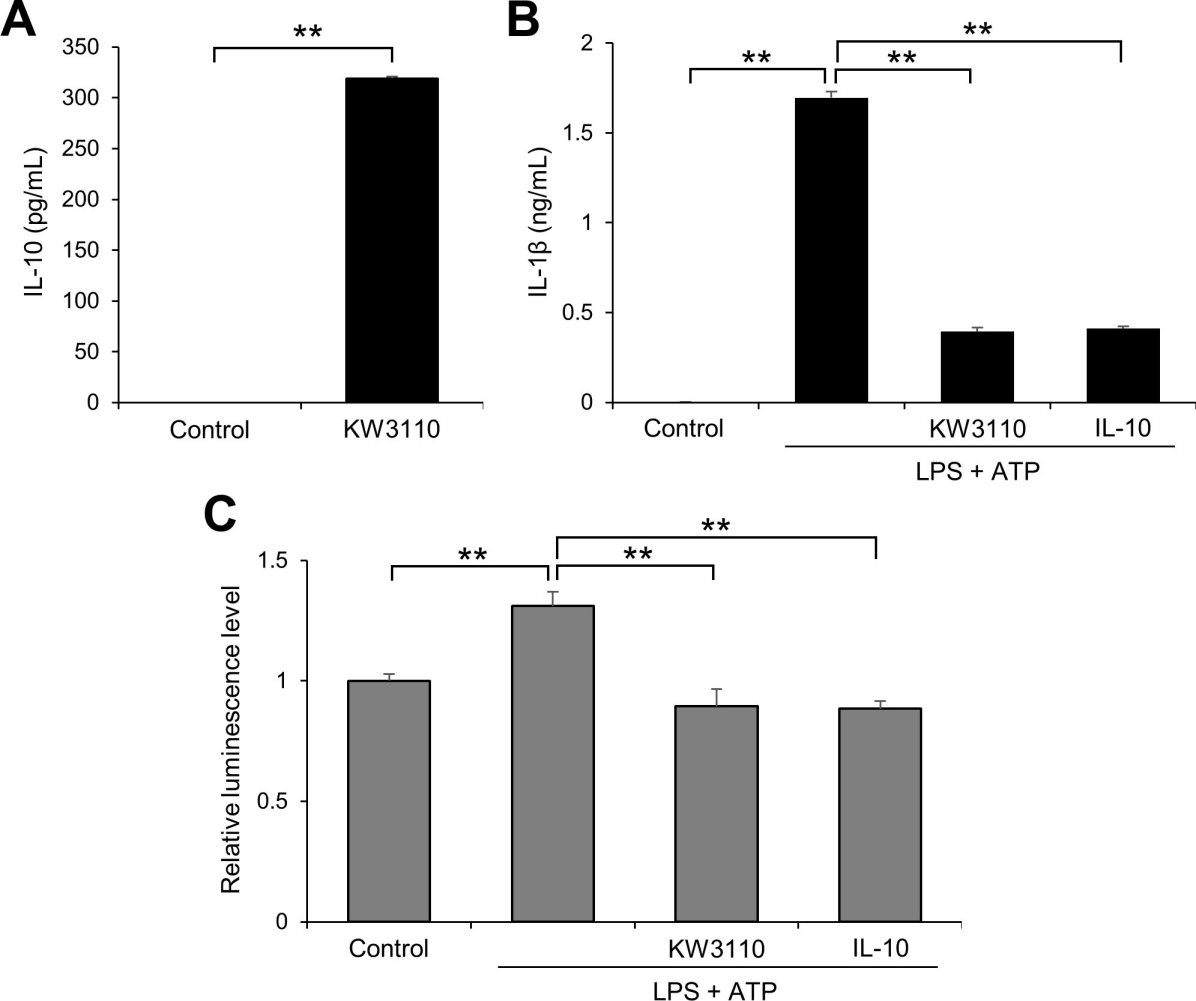
KW3110 treatment induces IL-10 production and suppresses IL-1β production and caspase-1 activity in human monocytes. (A-C) Human monocytes were treated with KW3110 (100 μg/mL) for 24 h. IL-10 levels in the supernatant were measured by ELISA (A). The cells were then treated with IL-10 (1 μg/mL) and LPS (10 μg/mL) for 4 h and ATP (2 mM) for 1 h. IL-1β levels in the supernatant were measured by ELISA (B) and the supernatant was analyzed by Caspase-Glo 1 inflammasome assay (C). Values indicate means ± SEM (triplicate data). Statistical differences were analyzed by unpaired *t*-tests for (A) and ANOVA followed by Tukey’s test for (B, C) (**, *P* < 0.01).

Finally, we investigated whether KW3110 treatment suppresses caspase-1 activity and IL-1β production through the IL-10 signaling pathway in human monocytes, isolated from human peripheral blood mononuclear cells used for inflammasome assay, as well as J774A.1 cells. KW3110 treatment significantly increased IL-10 levels in the supernatant from human monocytes when compared with the control (*P* < 0.001; Fig 6A). While LPS/ATP stimulation significantly increased IL-1β levels in cell supernatants (*P* < 0.001; Fig 6B), KW3110 pre-treatment significantly suppressed the production of IL-1β (*P* < 0.001; Fig 6B). Furthermore, caspase-1 activity levels were significantly increased by LPS/ATP stimulation of human monocytes (*P* = 0.010; Fig 6C), and significantly suppressed by KW3110 pre-treatment (*P* = 0.001; Fig 6C). Similar to KW3110 pre-treatment, recombinant human IL-10 protein treatment significantly suppressed IL-1β production and caspase-1 activation in LPS/ATP-stimulated human monocytes (*P* < 0.001; Fig 6B) (*P* = 0.002; Fig 6C).

## Discussion

Previously, a specific strain of LAB, KW3110, was shown to activate M2 macrophages and have anti-inflammatory effects, including suppression of IL-1β production, but the underlying mechanism was not well understood. Our study demonstrated that KW3110 suppressed LPS/ATP stimulation-induced caspase-1 activation and IL-1β production by promoting IL-10 production in murine macrophages. In addition, we showed that the mechanism of action may be similar in human monocytes.

**Fig 7:**
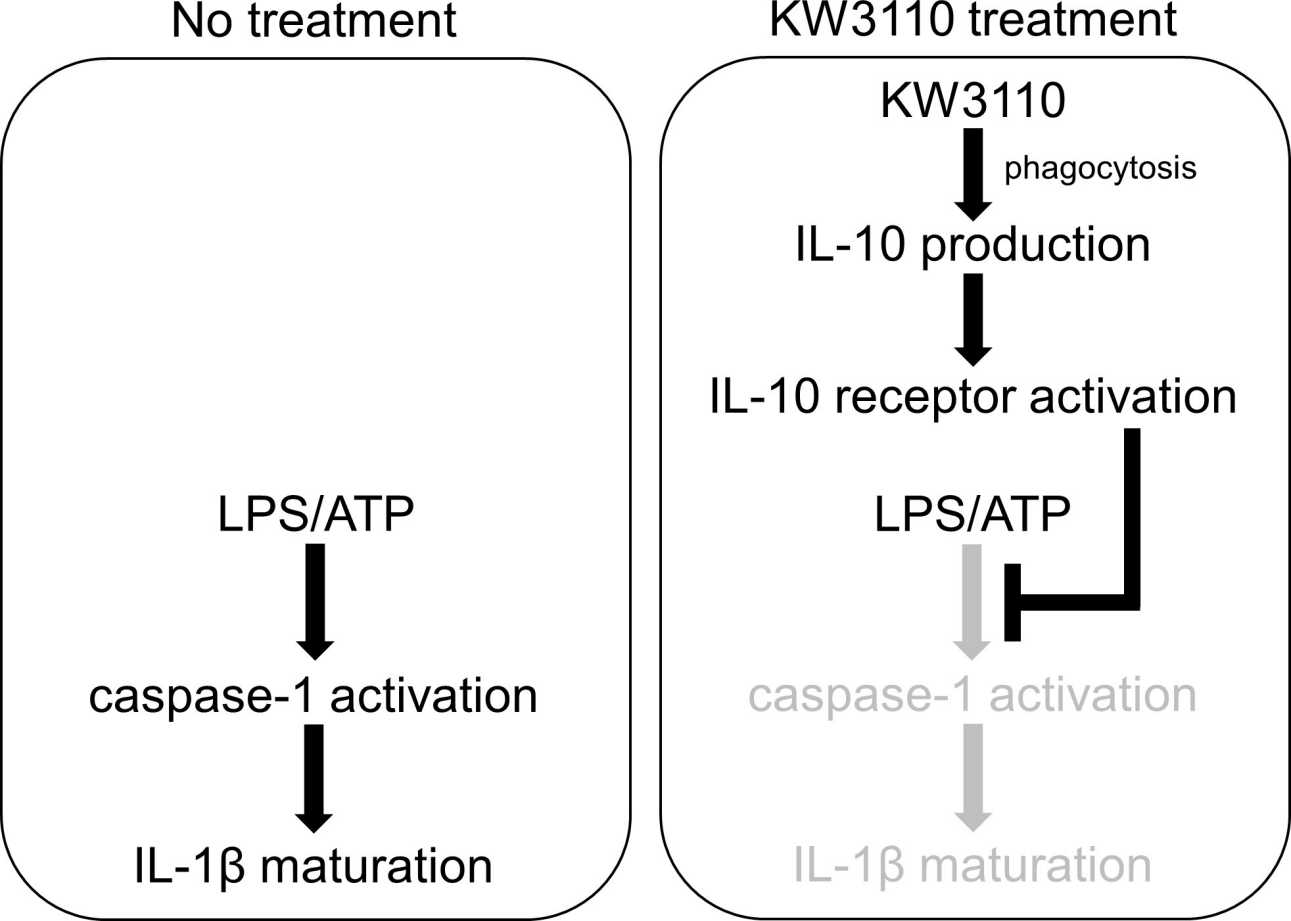
A signaling diagram of anti-inflammatory mechanism of KW3110. While LPS/ATP stimulation induces caspase-1 activation and IL-1β maturation in non-treated J774A.1 cells, KW3110 treatment suppresses these inflammatory responses in the cells through promoting IL-10 production.

In this study, some of the molecules released from KW3110-stimulated J774A.1 cells were shown to suppress IL-1β production in LPS/ATP-stimulated cells (Fig 1B). IL-10 production was upregulated in the supernatant from KW3110-stimulated cells (Fig 2A), and knockdown of IL-10 signaling components using siRNAs inhibited the suppressive effect of KW3110 on IL-1β production in LPS/ATP-stimulated cells (Fig 2E). In addition, IL-10 production was upregulated in the supernatant from KW3110-stimulated human monocytes (Fig 6A), and both KW3110 and recombinant IL-10 protein treatments suppressed IL-1β production in LPS/ATP-stimulated cells (Fig 6B). IL-10 is a representative anti-inflammatory cytokine and is reported to be involved in inhibition of IL-1β production [23–25], which is consistent with our results shown in Fig 2B. KW3110 was previously reported to activate macrophages by interacting with gut immune cells and promoting cytokine production, including IL-10 [10, 13, 20]. It has also been reported that KW3110 suppresses the level of pro-inflammatory cytokines such as IL-1β in the serum of aged mice or the retina of blue-light exposure-induced mice [13, 14]. These results suggest that KW3110 ameliorates IL-1β production in inflammatory-stressed immune cells through the IL-10 signaling pathway in both mice and humans (Fig 7).

The effects of KW3110 on both induction of IL-10 production and suppression of IL-1β production in J774A.1 cells were inhibited by cytochalasin D, a phagocytosis blocker (Fig 3). These results are consistent with previous reports showing that microbes, including LAB, are detected and phagocytosed by immune cells such as macrophages, followed by production of pro- or anti-inflammatory cytokines [21, 22]. We suggest that KW3110 has anti-inflammatory effects which are dependent upon the phagocytosis process of macrophages. A variety of innate immune receptors including scavenger receptors, C-type lectins and Toll-like receptors are expressed in the cell membrane of macrophages and play a key role in recognizing some of the structures on the bacterial surface, thus facilitating phagocytosis [26, 27]. Therefore, the anti-inflammatory effect of KW3110 on macrophages might be achieved through recognition of KW3110 surface structures by macrophage receptors and the phagocytosis process.

KW3110 treatment suppressed mature IL-1β expression in the supernatants from LPS/ATP-stimulated J774A.1 cells, but not pro-IL-1β expression in the cell lysates (Fig 4A), indicating that KW3110 may affect the maturation process of IL-1β. IL-1β maturation is promoted by the activity of caspase-1, which is an active component of inflammasome complexes [28]. In our study, KW3110 treatment reduced caspase-1 activation in the supernatant from LPS/ATP-stimulated J774A.1 cells and this effect was inhibited by transfection with siRNAs for *IL-10* or *IL-10 receptor α* (Figs 4B and 5A). In addition, IL-10 treatment reduced caspase-1 activation in the supernatant from LPS/ATP-stimulated cells (Fig 5B), which is supported by previous reports showing that IL-10 is involved in the inhibition of inflammasome activation [29, 30]. Inflammasome activation is promoted by the production of mitochondrial reactive oxygen species and oxidized mitochondrial DNA derived from mitochondrial dysfunction [31, 32]. The effect of IL-10 on inhibiting inflammasome activation was suggested to be dependent on mitophagy induction, which eliminates dysfunctional mitochondria; KW3110 might induce mitophagy through the IL-10 signaling pathway [25]. In addition, these results suggest that KW3110 may mitigate inflammasome activation in inflammatory-stressed macrophages through the IL-10 signaling pathway.

In conclusion, our results demonstrate that KW3110 suppresses LPS/ATP stimulation-induced caspase-1 activation and IL-1β production by promoting IL-10 production in mouse and human immune cells. The suppressive effect of KW3110 on caspase-1 activation is a novel anti-inflammatory mechanism of LAB. These findings are expected to contribute to constructing future preventive strategies for inflammation-related disorders using food ingredients.

## Supporting information

S1 Fig Raw data of western blot from Fig 4A. (TIF)

S2 Fig Raw data of western blot from Fig 4B. (TIF)
