# Supplementary figures and images for: Lactic acid bacterium, Lactobacillus paracasei KW3110, suppresses inflammatory stress-induced caspase-1 activation by promoting interleukin-10 production in mouse and human immune cells
Source: PLoS One. 2020 Aug 17;15(8):e0237754. doi: 10.1371/journal.pone.0237754 (PMC7430740; doi:10.1371/journal.pone.0237754)

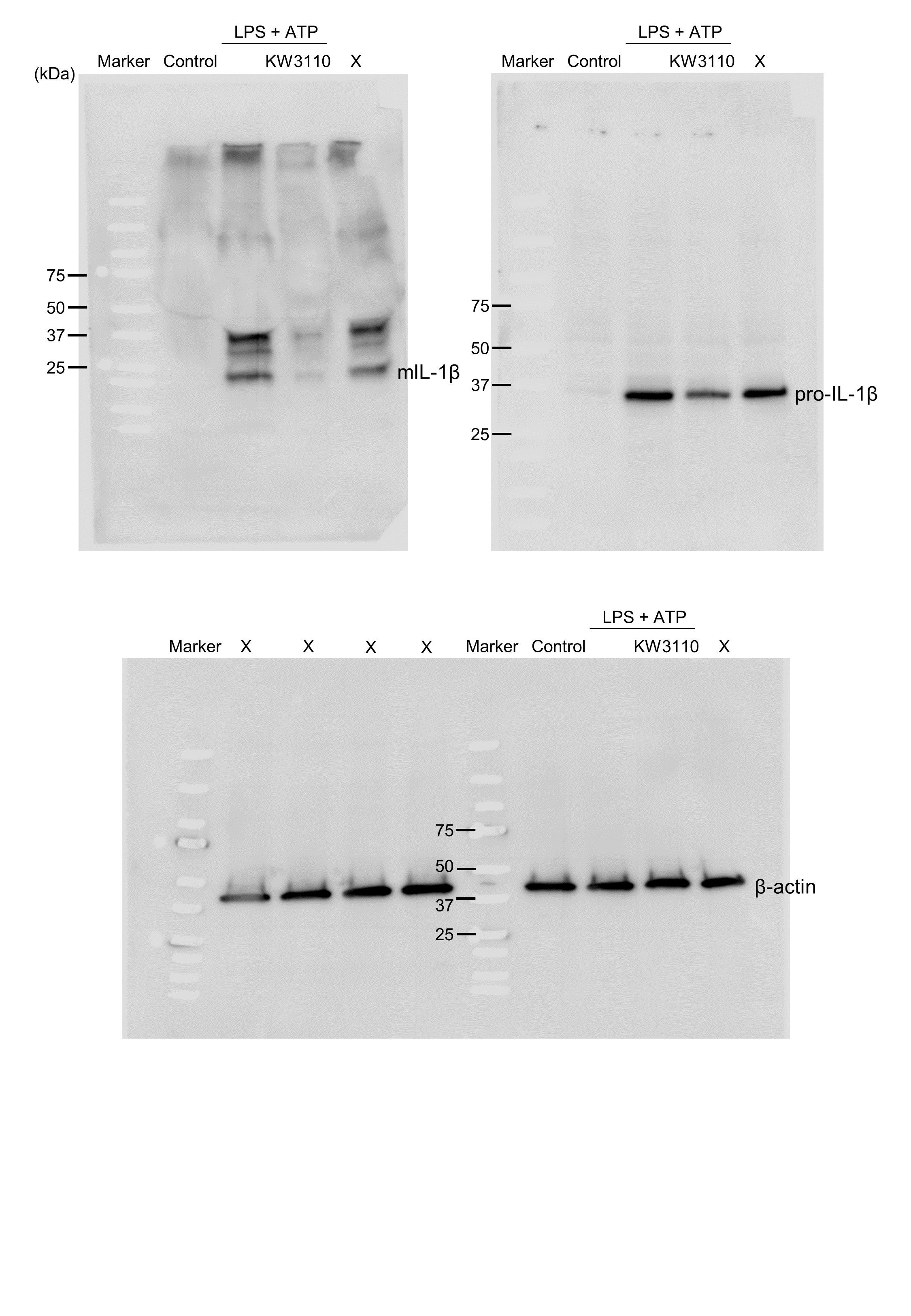

Supplement: S1 Fig — (TIF) [file pone.0237754.s001.tif]

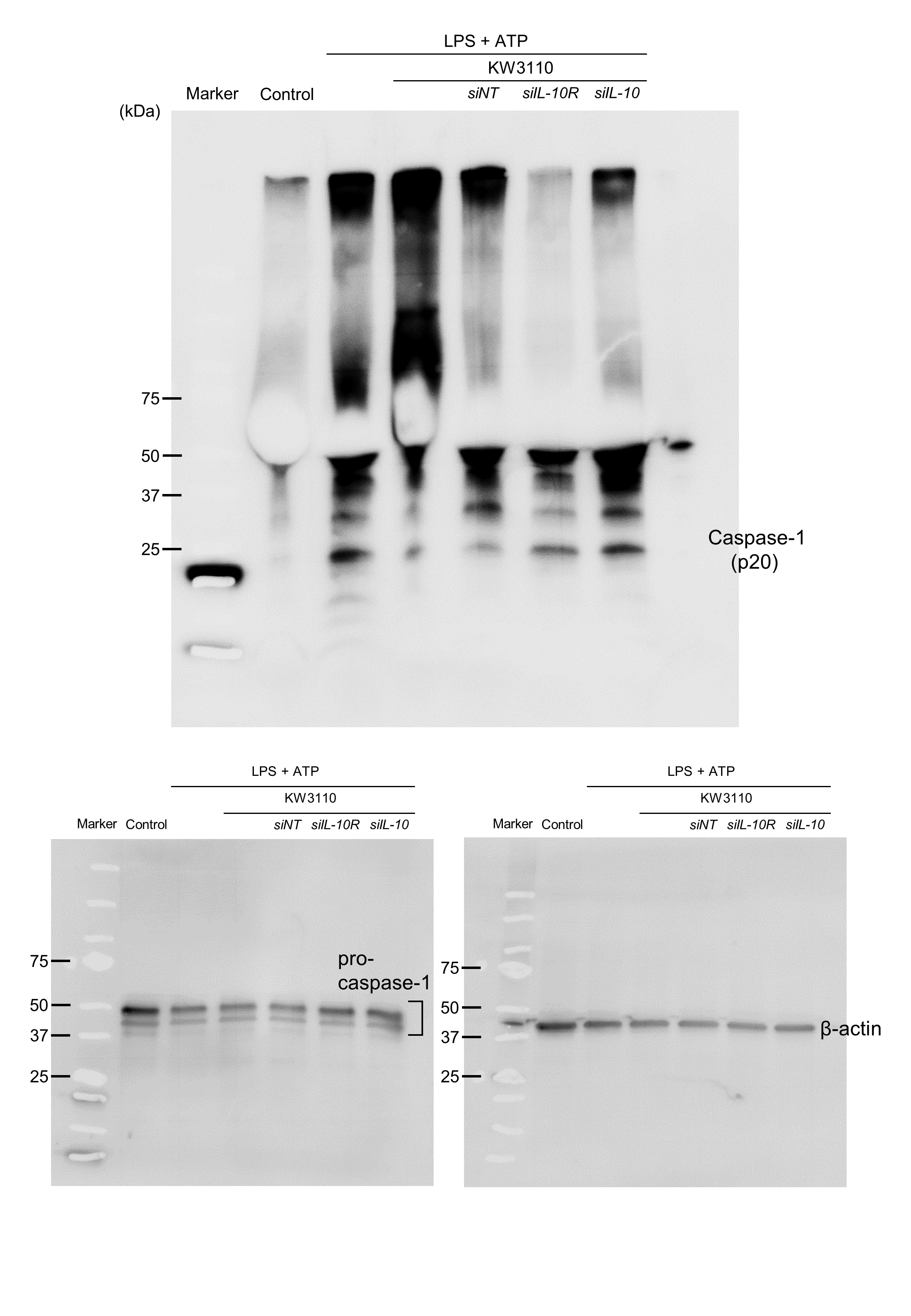

Supplement: S2 Fig — (TIF) [file pone.0237754.s002.tif]
